# Improving Access for Students From Widening Participation Backgrounds Applying to Medical School: A Quality Improvement Project

**DOI:** 10.7759/cureus.94805

**Published:** 2025-10-17

**Authors:** Shruthi Mankal, Faisa Ali, Chris Jacobs

**Affiliations:** 1 Medical Education, Great Western Hospital NHS Foundation Trust, Swindon, GBR; 2 Medical Education, University of Oxford, Oxford, GBR; 3 Faculty of Life Sciences and Medicine, King's College London, London, GBR; 4 Psychology, University of Bath, Bath, GBR

**Keywords:** confidence, educational intervention, interview skills, medical school application, preparedness, quality improvement project, widening access, widening participation, work experience

## Abstract

Introduction

Students from widening participation (WP) backgrounds - including those attending non-selective state schools, from lower socioeconomic groups, and from underrepresented ethnic groups - have a disproportionately low number of applications to medical school in the United Kingdom. Evidence suggests that such students have limited knowledge of the medical school application process, less support from teachers, and fewer contacts to access work experience. This quality improvement project (QIP) aims to identify challenges faced by students from WP backgrounds when applying to medical school, by implementing a pilot WP programme to address these challenges and improve their confidence and preparedness.

Methods

Challenges were first identified through a focus group (n = 4) and an anonymised pre-intervention survey. A WP programme was subsequently developed in line with student feedback and delivered at a district general hospital to 20 participants aged 16-18. Programme components included talks on the application process, hospital-based work experience, and interview practice. A mixed-methods evaluation compared pre-intervention (n = 16) and post-intervention (n = 18) questionnaires, with paired responses tracked using anonymous identifiers. Self-reported changes in confidence in the application process and preparedness for interviews (via Likert scale: 1 = not confident/prepared at all, 5 = very confident/prepared) were analysed using the Wilcoxon signed-rank test. McNemar’s test assessed directional changes in the number of application-strengthening themes identified. Thematic analysis of free-text responses identified perceived benefits pre-intervention, what applicants found most useful, and further support needs post-intervention.

Results

Following the pilot WP programme, there was a statistically significant improvement in confidence in the application process (n = 13, p = 0.003, r = 0.79), with median scores rising from 3 (IQR 3-4) to 4 (IQR 4-5), and in feeling prepared for medical school interviews (n = 11, p = 0.003, r = 0.90), with medians increasing from 2 (IQR 1-3) to 3 (IQR 3-4). A greater breadth of application-strengthening themes was reported post-intervention, with a statistically significant increase in students identifying exposure to life as a doctor as a strength (from 3 to 12, p = 0.004). Similarly, thematic analysis found that students noted clinical and career exposure to be the most useful aspects of the programme, followed by interview preparation and professional identity development. The small sample limited power, preventing subgroup analysis, while limited ethnic diversity, single-centre study design, and self-reported outcomes limit generalisability.

Conclusions

This QIP found that a pilot WP programme, designed with student input, improved students’ confidence and knowledge in navigating the medical school application process by strengthening various domains, including their confidence throughout the process, exposure to life as a doctor, and readiness for interviews. Such initiatives can be easily adopted by other centres. Future iterations should incorporate more work experience and long-term mentorship to better support underrepresented applicants, prioritise recruiting students from underrepresented ethnic backgrounds, and include longitudinal follow-up. Further attention is required to ensure WP students retain support within medical school.

## Introduction

Admission to medical school in the United Kingdom (UK) is characterised by persistent disparities in applicant demographics and success rates. Widening participation (WP) refers to efforts to increase access to higher education for students from underrepresented or disadvantaged backgrounds, including those from lower socioeconomic groups, state-funded schools, and certain ethnic groups [1]. A large retrospective analysis of candidates sitting the UK Clinical Aptitude Test (UCAT), a prerequisite for UK undergraduate medical school entry, demonstrated that 80% of applicants originated from higher socioeconomic groups [1]. The proportion of applicants from independent schools was nearly four times higher than expected, whereas the ratio for those from state-funded schools was only 0.78 of what is expected relative to their representation in the wider population. Students attending private schools have higher acceptance rates into medicine, independent of academic ability, suggesting that additional factors may enhance their applications [2]. Evidence highlights that secondary school students from WP backgrounds face barriers due to limited knowledge of the medical school application process and less support from teachers knowledgeable about the application process [3]. Furthermore, inequalities within WP groups are evident, with some students able to secure work experience placements through personal networks, while others lack such opportunities. Representation also varies across ethnic groups, as UK medical school admission ratios differ tenfold between applicants identifying as Asian students (over-represented) and those identifying as White students (under-represented) [4].

WP initiatives seek to address these inequities by providing targeted support to students from disadvantaged backgrounds, enabling greater access to higher education opportunities [1]. Previous interventions have shown promise, with a UK-based, medical student-led WP initiative improving participants’ understanding of topics assessed in the application process [5]. However, these initiatives were largely designed around faculty-identified priorities rather than being informed by the specific needs and perspectives of students themselves. This highlights a gap in student-led interventions that are co-designed with, and tailored to, the needs of WP students. This quality improvement project (QIP) aims to identify challenges faced by students from WP backgrounds when applying to medical school, and to implement a pilot WP programme to address these challenges - ultimately aiming to improve confidence and preparedness for applying to medical school.

## Materials and methods

Theoretical framework and study design

**Figure 1:**
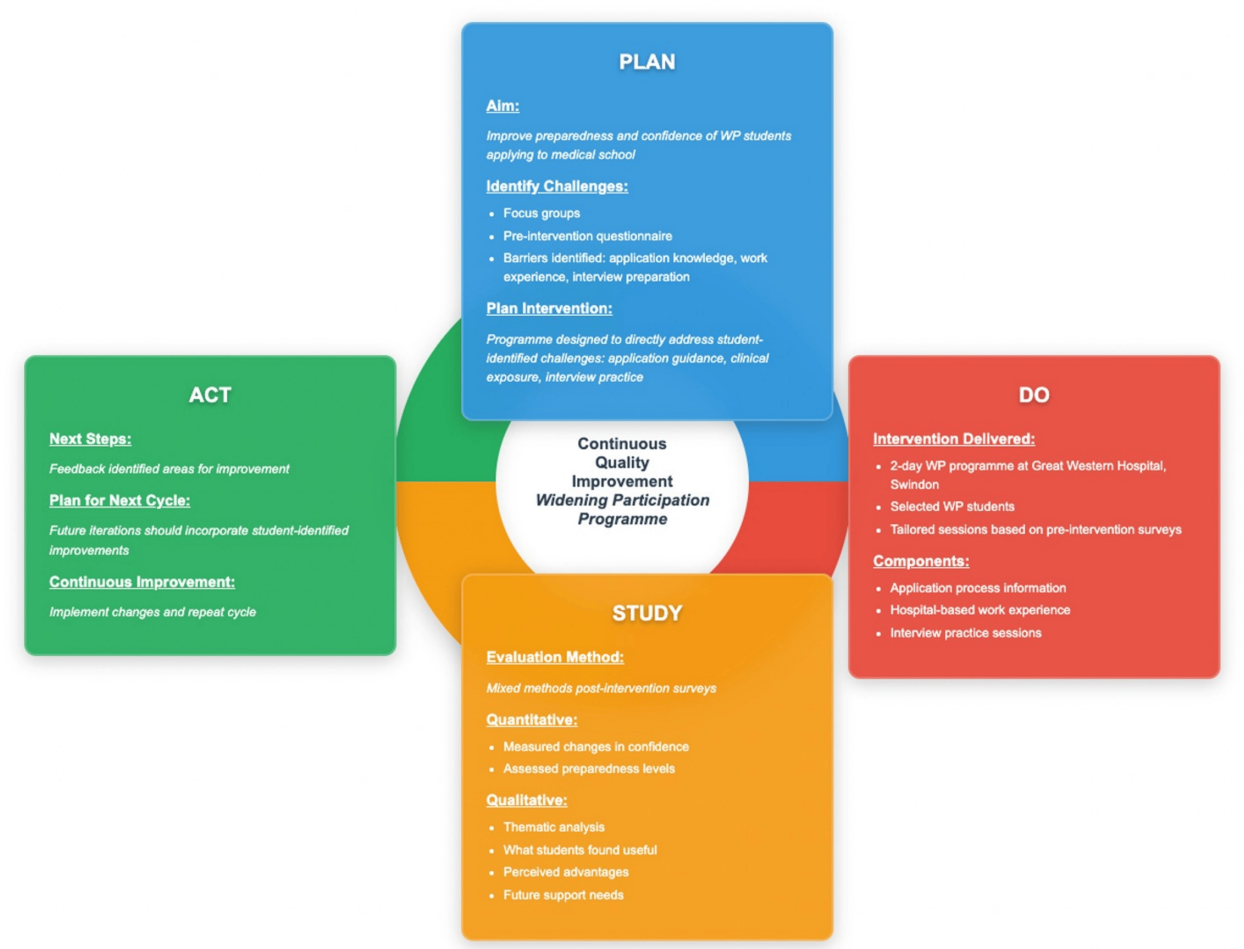
Plan-Do-Study-Act (PDSA) cycle implementation for the widening participation quality improvement project.

This QIP followed the Plan-Do-Study-Act (PDSA) framework [6], with the study design outlined in Figure 1. The results of the first PDSA cycle are included, with the report prepared in accordance with the Standards for Quality Improvement Reporting Excellence in Education (SQUIRE-EDU) guidelines (Appendix A) [7]. A mixed-methods approach combined quantitative self-reported data collected pre- and post-intervention with thematic analysis of qualitative free-text responses. A 20-minute focus group (n = 4) was conducted by two researchers to explore students’ perspectives. Participants were recruited from students expressing interest in attending the programme or who had attended an open evening explaining the WP initiative. The focus group followed a semi-structured guide (Appendix B) and was audio-recorded, anonymised, and transcribed. Transcripts were reviewed to identify common themes (Appendix C). Responses to a pre-intervention survey from successful applicants from WP backgrounds informed programme design. Post-programme surveys evaluated outcomes and identified opportunities for improvement, which we will state as actionable steps for Cycle 2.

Participants

Twenty students who are in sixth form from WP backgrounds participated in this pilot programme. Recruitment was conducted through flyers sent to local schools and an open evening held at Great Western Hospital, Swindon, where students interested in pursuing a career in medicine could find out more about the WP programme. All students were invited to complete a standardised application form. Eligibility criteria included being aged 16-18 years and attending a state-funded school. Initial priority was given to students attending non-selective state schools; however, the inclusion criteria were later expanded to include students attending selective state schools, due to the availability of remaining places.

Descriptive results from the anonymised pre-intervention demographics survey (n = 19) are summarised in Table 1. As the project evaluated an educational intervention for school students aged ≥16, without involving patients or clinical staff providing direct patient care, it was classified as an educational QIP. All participants provided informed consent, and data were collected anonymously.

**Table 1:** Demographics data of participants.

Demographic Category	Subgroup	N	%
Gender	Female	14	73.7
	Male	4	21.1
	Non-binary	0	0
Type of School Attended	Non-selective State School	17	89.5
	Selective State (Grammar) School	2	10.5
	Independent/Private School	0	0
Ethnicity	White - British or Other Students	8	42.1
	Asian/Asian British - Indian or Pakistani or Other Students	7	36.8
	Mixed/Multiple Ethnic Groups	2	10.5
	Black or Black British - African Students	2	10.5

Intervention

This two-day WP programme was conducted at Great Western Hospital, Swindon. On the first day, resident doctors gave talks on the medical school application process, including timelines and the key components of the application. Students attended clinical skills workshops and participated in simulated assessments of unwell patients. Mock panel interviews were conducted to provide interview practice. The second day focused on hospital work experience, with students assigned a full day at either a medical, surgical, or urgent care department at this district general hospital.

Data collection

Pre- and post-intervention data were collected anonymously using online questionnaires (Appendices D and E). The two main assessment criteria for UK medical school entry involve the candidate’s initial application (encompassing the UCAT) exam score and personal statement) and interviews. Hence, confidence scores regarding the medical school application process and interview preparedness were measured using a five-point Likert scale (1 = lowest, 5 = highest), before and after the programme.

A checklist-style question, “What advantages do you feel strengthen your medical application?” was administered pre- and post-programme, allowing participants to select from a predefined list of potential advantages. Free-text responses were also collected to identify the support participants perceived as most useful pre-intervention, and what they found most valuable post-intervention.

Analysis

Anonymous identifiers were used to maintain confidentiality but enable paired analysis of pre- and post-intervention responses. Due to the ordinal nature of Likert scale responses, non-parametric tests were used to assess changes in confidence in knowledge of the application process and preparedness for interviews. Normality was assessed using the Shapiro-Wilk test, which indicated non-normal distribution. Consequently, changes in paired responses were analysed using the Wilcoxon signed-rank test, with effect sizes (r) calculated to quantify the magnitude of change. To assess whether participants identified a broader range of application-strengthening factors after the programme, the number of distinct coded themes in paired pre- and post-intervention responses was calculated and compared using the Wilcoxon signed-rank test. Additionally, McNemar’s test was applied to assess directional changes in theme count. All statistical analyses were performed using R software (v4.5.1; R Foundation for Statistical Computing, Vienna, Austria).

Qualitative data from free-text survey responses underwent reflexive thematic analysis following Braun and Clarke’s six-step method [8]. This involves initial familiarisation with the data, before generating initial codes, searching for themes, reviewing, defining, and naming the themes, and writing a final report. Two researchers - one being a medical educator and the other having a background working in widening access to higher education - independently coded the data, then collaboratively identified common themes through an inductive approach and iterative process.

## Results

There were 16 responses to the pre-intervention questionnaire and 18 responses for the post-intervention questionnaire after removing duplicates (pre-programme n = 1 and post-programme n = 2). 

Quantitative pre- and post-intervention comparisons

For matched results, there were 14 participants after removing four unmatched responses (due to lack of post-programme responses) and two duplicates. 

Confidence in the Application Process and Medical School Interviews

**Figure 2:**
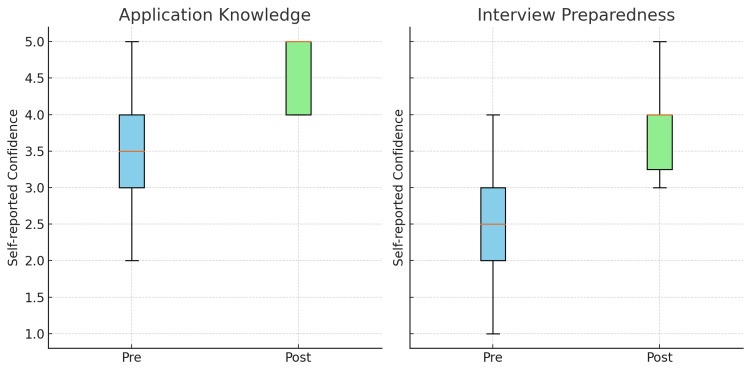
Boxplots illustrating the differences in self-reported confidence of participants in application knowledge (left) and interview preparedness (right), pre- and post-intervention. Error bars indicate interquartile ranges.

There was a statistically significant increase in participants’ confidence regarding their knowledge of the application process (W = 5.0, Z = -2.87, n = 13, p = 0.003), with a large effect size (r = 0.79). Median scores increased from 3 (IQR: 3-4) before the programme to 4 (IQR: 4-5) afterwards, as demonstrated in Figure 2. Similarly, participants reported greater preparedness for medical school interviews following the intervention (W = 0.0, Z = -2.98, n = 11, p = 0.003), with a large effect size (r = 0.90). Median scores rose from 2 (IQR: 1-3) pre-programme to 3 (IQR: 3-4) post-programme, as seen in Figure 2. These findings suggest that the programme had a meaningful and substantial positive impact on participants’ perceived readiness for the medical school application process.

Advantages of Strengthening Medical School Application

Participants reported a significantly greater number of distinct themes post-programme (median = 3, IQR: 2-4) compared to pre-programme (median = 2, IQR: 1-3), indicating an expanded awareness of factors that strengthen a medical application (Wilcoxon signed-rank test, W = 5.5, p = 0.003). Moreover, McNemar’s exact test confirmed a statistically significant shift in thematic breadth: 12 participants identified more themes after the programme than before, while only one showed a decrease (p = 0.003). These findings suggest that the programme not only influenced the frequency of specific themes, such as exposure, but also broadened participants’ overall understanding of what constitutes a strong medical application.

Participants’ open-ended responses describing perceived advantages for their medical school applications were thematically coded into four categories: confidence, knowledge of the application process, exposure to life as a doctor, and connections. McNemar’s tests were used to evaluate whether the frequency of each theme changed significantly following the intervention. Mentions of confidence increased from 7 participants pre-programme to 11 post-programme; however, this change was not statistically significant (χ²(1, n = 14) = not significant, p = 0.45). In contrast, the number of participants identifying exposure to life as a doctor as a key advantage rose markedly from 3 to 12. This increase was statistically significant, indicating that the programme substantially enhanced participants’ awareness of clinical experience as an important application strength (p = 0.004). Mentions of connections remained relatively stable, increasing only slightly from one to four participants, with no statistically significant change observed (p = 1.00).

Qualitative thematic analysis

According to the six steps outlined by Braun and Clarke’s guidelines for performing a thematic analysis [8], key themes and sub-themes were generated for free-text responses to two questions in the pre-intervention and post-intervention questionnaires.

What Type of Support Would Be Most Helpful for You?

**Figure 3:**
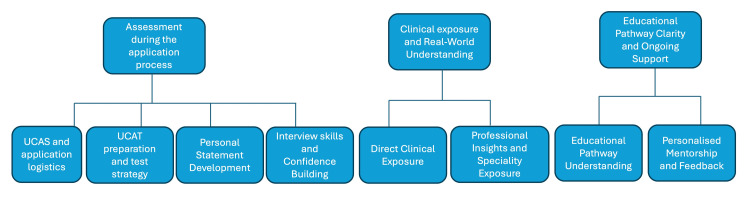
Key themes (first row) and sub-themes (second row) generated from student responses to the question: “What type of support would be most helpful for you (in applying to medical school) - give at least TWO answers.” UCAS, Universities and Colleges Admissions Service; UCAT, UK Clinical Aptitude Test

Participants in the pre-intervention questionnaire were asked: “What type of support would be most helpful for you - give at least TWO answers.” This questionnaire had 16 responses, after removing one duplicate, from which three broad themes were generated: “assessment during application process,” “clinical exposure and real-world understanding,” and “educational pathway clarity and ongoing support.” Each key theme had further sub-themes, as outlined in Figure 3, with the number of responses discussing each sub-theme outlined in Table 2. 

**Table 2:** Themes and sub-themes generated from thematic analysis of the free-text responses to the question: “What type of support would be most helpful for you (in applying to medical school) - give at least TWO answers.” “Number of references” refers to the total number of participants who mentioned each sub-theme in their free-text responses, with “% of participants” indicating the percentage of total participants who mentioned each sub-theme. UCAS, Universities and Colleges Admissions Service; UCAT, UK Clinical Aptitude Test

Theme	Sub-theme	Number of References (Total Number of Participants: n = 16)	% of Participants
Assessment During the Application Process	UCAS and Application Logistics	5	31%
	UCAT Preparation and Test Strategy	3	19%
	Personal Statement Development	3	19%
	Interview Skills and Confidence Building	8	50%
Clinical Exposure and Real-World Understanding	Direct Clinical Experience	6	38%
	Professional Insights and Speciality Exposure	2	13%
Education Pathway Clarity and Ongoing Support	Educational Pathway Understanding	2	13%
	Personalised Mentorship and Feedback	4	25%

Assessment during the application process: Of the 16 responses, there were 19 mentions of aspects of the theme “assessment during the application process” and its sub-themes. The most popular sub-theme by far was “interview skills and confidence building,” with eight individuals listing it as a type of support that would be most helpful for them. Another popular sub-theme was “UCAS and application logistics,” with five individuals listing it as their most helpful type of support. Students reported that they needed “support in the application process” and guidance on “what I should do in terms of UCAS.”

Clinical exposure and real-world understanding: Clinical experience and exposure to specialities were another theme generated by student responses. There were eight total mentions in the responses, with six of the eight being categorised into the “direct clinical experience” sub-theme. Students felt they would benefit most from “having a clinical placement” and wanted “exposure to life as a doctor.” Two students also mentioned that clinical exposure would be helpful in providing them with professional insights and exposing them to different specialities.

Educational pathway clarity and ongoing support: Finally, students reported that they would find more information about the academic course itself and personalised mentorship useful. Four individuals mentioned that they would benefit from mentorship in various domains, including support with aspects of the application, the interview process, “constructive feedback,” and “personal and wellbeing support.”

What Did You Find Most Useful in This Programme?

**Figure 4:**
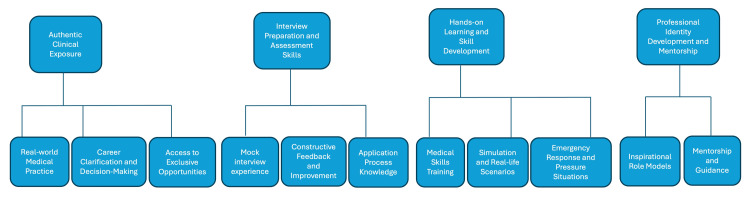
Key themes (first row) and sub-themes (second row) generated from student responses to the question: “What did you find most useful in this programme? Why?”

The same process was performed for the post-intervention questionnaire. Students answered the question: “What did you find most useful in this programme? Why?” This yielded 18 free-text responses, after removing two duplicates. From these responses, four key themes were generated, including “clinical and career exposure,” “interview preparation and assessment skills,” “hands-on learning and skill development,” and “professional identity development and mentorship.” The key themes and their sub-themes are outlined in Figure 4, with the number of responses to each sub-theme outlined in Table 3.

**Table 3:** Themes and sub-themes generated from thematic analysis of the free-text responses to the question: “What did you find most useful in this programme? Why?” “Number of references” refers to the total number of participants who mentioned each sub-theme in their free-text responses, with “% of participants” indicating the percentage of total participants who mentioned each sub-theme.

Theme	Sub-theme	No. of References (Total Number of Participants: n = 18)	% of Participants
Clinical and Career Exposure	Real-World Medical Practice	12	67%
	Career Clarification and Decision-Making	6	33%
	Access to Exclusive Opportunities	3	17%
Interview Preparation and Assessment Skills	Mock Interview Experience	7	39%
	Constructive Feedback and Improvement	5	28%
	Application Process Knowledge	2	11%
Hands-On Learning and Skill Development	Medical Skills Training	1	6%
	Simulation and Real-Life Scenarios	4	22%
	Emergency Response and Pressure Situations	2	11%
Professional Identity Development and Mentorship	Understanding Professional Roles	7	39%
	Inspirational Role Models	2	11%
	Mentorship and Guidance	1	6%

Clinical and career exposure: This theme accounted for the majority of student responses to the post-intervention questionnaire, with 12 individuals reporting the sub-theme “real-world clinical practice” as the most useful aspect of the programme - by far the most of any sub-theme. Students reported that the “clinical placement was incredibly useful,” as it showed “what it is like to be a doctor,” instead of just “learning and hearing about it.” Students also found that they had greater “career clarification” and “support with decision-making,” with six individuals identifying this sub-theme as what they found most useful. Students also found the intervention useful in that it was an exclusive experience.

Interview preparation and assessment skills: There were 11 total mentions sorted into this theme, across the sub-themes: “mock interview experience,” “constructive feedback and improvement,” and “application process knowledge.” The most responses were recorded for “mock interview experience,” which had seven responses in total. Students reported that they found the mock interview most useful, as it “is the first one” they’ve done “specifically for Medicine.”

Professional identity development and mentorship: There were 10 overall mentions of “professional identity development and mentorship” across this theme, with the majority falling under the sub-theme “understanding professional roles.” Seven students referenced this as the most useful part of their experience, mentioning that “it was a way to experience real life as a doctor instead of just learning and hearing about it,” and that it was “incredibly useful,” as they did not really “know what you’re getting yourself into unless you experience it.”

## Discussion

This pilot study was well-represented in the schooling background of participants: 17 of the 19 who completed the survey were from non-selective state school backgrounds, highlighting that the study successfully targeted students of the correct schooling background when aiming to widen participation. The programme also had clear impacts across three domains: students’ exposure to life as a doctor, confidence in the application process, and interview skills. Notably, “exposure to life as a doctor” showed a statistically significant increase in student responses, from 3 to 12 (p = 0.004), reflecting how the programme provided access to experiences that are otherwise difficult to obtain without personal connections [3,9]. Confidence improved significantly in matched pre- and post-intervention responses [10], while interview skills and preparation were consistently valued, with mock interview practice helping to address recognised gaps for WP students [3]. These domains emerging as those most strongly impacting students across both the quantitative and qualitative analyses is of note; these qualities often constitute the “silent advantages” of students from more privileged backgrounds. By levelling the playing field in these areas, the programme addressed barriers that disproportionately affect applicants from underrepresented groups.

While it has historically been the case that students from minority ethnic backgrounds are less likely to be accepted into medical school - with McManus et al. establishing this in their seminal paper [11] - this is no longer the case. Indeed, as previously outlined, students from certain ethnic minority backgrounds are over-represented as medical school applicants [4]. This refers specifically to students from Asian backgrounds, who were the most over-represented; there was a 600-fold difference in admission ratios between Asian students of the highest social class and Black students of the lowest. This demonstrates that the puzzle of equitable admissions is multi-layered and no longer an issue of ethnic minority groups as a whole being underrepresented [12]. Rather, mobility is being seen within particular ethnic groups, and, more importantly, among members of these ethnic groups with the highest socioeconomic statuses [4]. Evidence consistently indicates that socioeconomic status has a profound impact on the likelihood of admission of students; social gradients can be generated for students who have been accepted into medical school, with the highest proportions coming from the most affluent postcodes and the lowest from the least affluent [1].

It is with this context that the results of our study should be considered. Most students were from a white background (eight), followed closely by students from a South Asian background (seven), compared to only two students from a Black or Black African background. Thus, while the intervention aimed to increase access to medical school, the extent to which it can be considered a comprehensive WP initiative is limited by the relatively low representation of minority ethnic groups. Future programmes of this type may benefit from prioritising the recruitment of students from ethnic backgrounds that remain underrepresented over others in the selection process. Increasing general awareness of the application process via the programme could also directly address historic weaknesses in the applications of students from minority ethnic backgrounds, including being “less qualified” and “applying later on” compared to their white counterparts [11]. Similar corrective measures should be taken to ensure there are students from diverse socioeconomic backgrounds, with students from the most deprived areas being prioritised in future cycles of the programme. Application forms could include questions on postcodes, with areas of highest need conferring priority to students most likely to be selected. Additionally, with the aim of increasing the scale of the programme in future cycles, travel bursaries may be beneficial to ensure student attendance.

Additional limitations of this study include the small sample size (n = 20), which restricts statistical power, preventing subgroup analyses - such as the impacts of ethnic minority status on domains of the intervention reported to be most useful - alongside gender and the type of school attended. The single-centre design restricts external validity, and the absence of a control or comparison group limits causal inference. Furthermore, outcomes were self-reported and measured immediately post-intervention, introducing potential response bias and limiting insight into sustained impact. 

Given these limitations, this study should be viewed as an exploratory pilot, with potential replicability at a greater scale in different regions of the UK to provide more robust, statistically well-powered, and generalisable data. Future iterations would benefit from multi-centre implementation, larger and more diverse cohorts, and longitudinal follow-up to assess sustained effects on application success rates. Changes for future cycles should be considered and acted upon in a similar manner to how participants were directly informed of the interventions in this programme. Student feedback will also directly inform its future iterations; as part of the post-intervention questionnaire, students were asked, “What further support would be most beneficial?” Students suggest that ongoing mentorship would improve the programme, further supported by evidence from another WP programme, which found an improved sense of community after establishing “Families,” by pairing prospective medical students with current ones [13]. Responses provided specific, constructive feedback for the next cycle of the WP programme, building on the precedent set in this study of addressing students’ needs by directly implementing their solutions in WP programmes for them and future prospective medical students.

## Conclusions

To conclude, this QIP provides preliminary evidence that a pilot WP programme, tailored to students’ needs, increases their knowledge of the application process across various domains - including their exposure to life as a doctor - and improves confidence in navigating applications and interview preparedness. This indicates its utility as a template for future WP programmes, suggesting student-informed approaches could be applied to support underrepresented students more effectively. Large, multi-centre studies are required to better ascertain the reproducibility of results and determine which subgroups of students benefit most. Moving beyond the scope of this study, similar techniques could be used to reduce the attrition rates of these same students in medical school by ensuring their needs are both addressed and well-supported - not just in applying, but also in retaining their positions. 

## Appendices

Appendix A

Standards for Quality Improvement Reporting Excellence in Education (SQUIRE-EDU) guidelines applied to this educational quality improvement project

**Table 4:** Standards for Quality Improvement Reporting Excellence in Education (SQUIRE-EDU) guidelines applied to this educational quality improvement project.

Text Section and Item Name	Mapping of Widening Participation (WP) Quality Improvement Project to SQUIRE-EDU Guidelines
Title and Abstract	
1. Title	Identifies the focus on WP and providing equity of opportunities for medical school applicants.
2. Abstract	Abstract: Structured to include problem description, available knowledge, rationale, methods, results, and conclusions. Keywords include a focus on education, confidence, preparedness and access to medical education.
Introduction: Why did you start?	
3. Problem Description	Limited opportunities for sixth-form students (particularly from underrepresented backgrounds) to access clinical work experience and support for medical school applications. Inequity in accessing support with medical school admissions. Barriers for WP students include limited knowledge of the application process, less teacher support, and unequal access to work experiences.
4. Available Knowledge	Prior analyses highlight socioeconomic and ethnic disparities. Previous interventions have improved the understanding of application topics. Limitations of prior work include that topics were determined by faculty, not student-perceived needs.
5. Rationale	Identifies persistent disparities in UK medical school admissions. Inequities not explained by academic ability suggest structural and social advantages. Barriers for WP students include limited knowledge of the application process, less teacher support, and unequal access to work experience.
6. Specific Aims	Justification: A student-informed intervention may better address real needs than faculty-led programmes. Aims: To identify challenges faced by students applying to medical school from WP backgrounds and implement an intervention to address them. To improve confidence and preparedness for applying to medical school.
Methods: What did you do?	
7. Context	Social/geopolitical influences partially addressed in the introduction: disparities in school type, socioeconomic status, and ethnic representation in medical school applications. Participant characteristics: Sixth-form students aged 16-18, from WP backgrounds. Detailed demographics: gender, school type, ethnicity. Organisational context: Conducted at a district general hospital (Great Western Hospital, Swindon). Availability of resident doctors, clinical skills workshops, and access to hospital departments affect the feasibility and content of the programme. Educational context: Pre-existing knowledge and support of participants: some have access to open evenings or prior information sessions; others have minimal guidance. Prior exposure to medical settings varies, influencing the benefit derived from work experience.
8. Intervention(s)	Follows the Plan-Do-Study-Act (PDSA) cycle framework. Intervention involved informative talks about the application process, clinical skills and simulation workshops (designed and run by resident doctors) and hospital work experience (where students were supervised by consultants and interacted with the multidisciplinary teams in their assigned department).
9. Study of the Interventions	Self-reported, anonymised pre- and post-intervention surveys.
10. Measures	Quantitative outcomes: Confidence in application process and interview preparedness (5-point Likert scale). Breadth of application-strengthening themes (predefined checklist). Qualitative outcomes: Free-text responses analysed thematically to identify perceived educational benefits.
11. Analysis	Quantitative: Shapiro-Wilk test confirmed non-normality; Wilcoxon signed-rank test for paired Likert responses; McNemar’s test for directional theme changes; effect sizes calculated. Qualitative: Reflexive thematic analysis using Braun & Clarke’s six-step method; independent coding by two researchers followed by collaborative theme identification.
12. Ethical Considerations	All participants provided informed consent. Data was collected anonymously. The project was classified as an educational quality improvement project without patient involvement.
Results: What did you find?	
13. Results	Process outlined in Figure 1. Quantitative outcomes reported: Confidence in application process: statistically significant improvement, median pre- vs post-scores, Wilcoxon test results, effect sizes. Preparedness for interviews: significant improvement with a large effect size. Breadth of application-strengthening themes: Wilcoxon signed-rank and McNemar’s tests indicate increased awareness. Qualitative outcomes reported: Thematic coding of free-text responses, of what students perceived to be most useful and what students found most helpful post-programme. Dat/Evidence: Statistical evidence provided: exact test values, n, p-values, effect sizes. Descriptive statistics: medians, IQRs, counts of mentions per theme. Tables/Figures referenced: Figures 2-3 for theme and sub-theme summaries. Comparison to Baseline: Pre- and post-intervention comparisons are consistently described: Paired analysis of Likert scores. Thematic coding comparisons highlight shifts in what students value or perceive as helpful. Reporting direction and magnitude of changes via paired analysis of Likert scale responses, with increases in confidence (increased from 3 to 4) and preparedness (increased from 2 to 3). Themes such as “exposure to life as a doctor” increased significantly (increased identification as an advantage from 3 to 12 participants). Sub-themes like “mock interview experience” or “real-world clinical practice” were highlighted as particularly impactful. Contextual Factors Affecting Outcomes: Demographics and prior experiences influence outcomes. Although the study had 20 participants, missing data were due to fewer respondents to the pre-intervention questionnaire (n = 16) and the post-intervention questionnaire. Unmatched responses resulted in 2 responses being excluded from quantitative analyses. The use of anonymous identifiers allowed the recognition of 3 duplicate responses for qualitative analyses, where each response was reviewed by both researchers and collated to remove duplicates.
Discussion: What does it mean?	
14. Summary	Summary of key findings: Observed improvements in confidence, preparedness for medical school interviews and understanding of the medical school application process. Quantitative and qualitative data reinforce the impact on specific domains such as exposure to life as a doctor and interview preparation.
15. Interpretation	Each major finding is interpreted in the context of existing knowledge/literature. Exposure to life as a doctor: highlighted as a unique benefit of the intervention, directly addressing gaps in real-world experience for WP students. Confidence: increased self-reported confidence linked to programme components (interviews, feedback, workshops). Interview skills: improvements explained by mock interview practice and constructive feedback. Findings are contextualised with prior studies. Compares current outcomes to previous WP interventions, highlighting alignment and unique contributions. Discussion explicitly links participant demographics to outcomes.
16. Limitations	Limitations are addressed. Sample characteristics: predominance of non-selective state school students but inclusion of selective state school students limits generalisability. Single-centre intervention restricts applicability to other settings. Small sample size resulted in insufficient power for subgroup analyses (ethnicity, gender, school type).
17. Conclusions	A pilot WP programme successfully improved confidence and preparedness. Insights could inform larger-scale interventions across multiple centres. Future programmes should be designed to include ongoing mentorship and incorporate longer, more varied work experience.
Other Information	
18. Funding	No funding was sought for the design, implementation, interpretation or reporting of this work. All staff involved in the intervention were volunteering their time.

Appendix B

Focus group prompts

**Table 5:** Focus group prompts. MacArthur ladder rating scale adapted from Goodman et al. [14].

Focus Group Prompts	Content
Case Scenario	Ahmed is 17 years old and aspires to study medicine. No one in his family has attended university. His family is supportive but unfamiliar with the application process. He sought advice at school, but it was generic and not tailored to his circumstances.
Case Discussion Questions for Students	1. What are the biggest challenges Ahmed is facing in his application?
	2. What would be the most helpful form of support for Ahmed to overcome these challenges?
MacArthur Ladder Rating	Participants viewed a 10-rung ladder and read: “Think of this ladder as showing where people stand in your neighbourhood. At the top are people who are best off - those with the most money, the best education, and the most respected jobs. At the bottom are people who are worst off - those with the least money, the least education, and the least respected jobs or no job. Where would you place yourself on the ladder in relation to other people in your neighbourhood? Tick the rung where you think you stand at this time in your life.” The MacArthur scale for neighbourhood social status was provided on paper for anonymous completion.
Prompts for Student Reflection	Prompt 1: When you think about applying to medical school, what are the immediate words that come to mind?
	Prompt 2: What do you perceive to be the biggest barriers to your entry into Medicine?
	Prompt 3: What do you think makes the most successful medical applicants successful?
	Prompt 4: What has the most useful support been that you’ve received in your application journey? Follow-up: Do you feel like anything impacts your answer to this?
	Prompt 5: Rate how prepared you feel in your application to Medicine on a scale of 1-10 (1 = completely unprepared, 10 = fully prepared and confident) and explain why.
	Prompt 6: If you were implementing a widening participation programme of your own, what would you prioritise including?

Appendix C

Focus group findings

**Table 6:** Focus group findings.

Theme	Key Findings From Participant Responses
Connections	When discussing the case with Ahmed, a lack of support on what to write in personal statements was key, and parents not having knowledge on the process was mentioned; "making more connections" was also listed as useful support for Ahmed.
Lack of Knowledge	Lack of knowledge was a significant barrier when asking participants about their biggest barriers to entry.
Lack of Confidence	Frequently came up as a concern for participants. It was cited as being one of their own barriers to entry into medicine. Participants also listed it as a reason for feeling unprepared in the process (when asked to rate how prepared they felt to apply). It was also mentioned as one of the advantages that make the most successful medical applicants successful.
Clinical Work Experience	Finding appropriate work experience was a common source of concern for participants. A participant called it the "most important" barrier when it came to entry into medicine, with the length of time taken to procure it also mentioned. A participant cited that the medical society at their school, giving them the tools to reflect on work experience, was one of the most useful forms of support in their application so far. Gaining clinical work experience and having patient interactions were mentioned when participants were asked what they prioritise, including in access schemes of their own.

Appendix D

Pre-programme questionnaire

**Table 7:** Pre-programme questionnaire.

Item No.	Question	Response Format
1	Please create an anonymous ID code using the first letter of your favourite colour and the last 3 digits of your phone number (e.g., if your favourite colour is blue and your phone number ends in 478, your code would be B478). This allows matching your answers with your feedback later.	Open text (anonymous ID code)
2	How confident do you feel in your knowledge of the application process?	Likert scale: 1 = Not confident at all; 5 = Very confident
3	How prepared do you feel for medical school interviews?	Likert scale: 1 = Not prepared at all; 5 = Very prepared
4	What are your challenges in applying to medical school? (Check all that apply)	Checkbox options: Finance (i.e., for paid opportunities/travel costs); Lack of connections; Lack of confidence/self-doubt; Lack of knowledge of application process; Lack of exposure to/knowledge of life as a doctor; Lack of mentorship/support; Lack of clinical placements; Other (please specify)
5	What advantages strengthen your medical school application? (Check all that apply)	Checkbox options: Finance (i.e., for paid opportunities/travel costs); Connections; Confidence in yourself/your skills; Knowledge of the application process; Exposure to/knowledge of life as a doctor; Mentorship/support; Clinical placements/shadowing; Other (please specify)
6	What type of support would be most helpful to you? Give at least TWO answers.	Free-text response

Appendix E

Post-programme questionnaire

**Table 8:** Post-programme questionnaire.

Item No.	Question	Response Format
1	Please state the same anonymous ID code as your pre-intervention questionnaire using the first letter of your favourite colour and the last 3 digits of your phone number (e.g., if your favourite colour is blue and your phone number ends in 478, your code would be B478).	Open text (anonymous ID code)
2	How confident do you now feel in your knowledge of the application process?	Likert scale: 1 = Not confident at all; 5 = Very confident
3	How prepared do you now feel for medical school interviews?	Likert scale: 1 = Not prepared at all; 5 = Very prepared
4	What advantages do you now feel strengthen your medical application? (Check all that apply)	Checkbox options: Finance (i.e., for paid opportunities/travel costs); Connections; Confidence in yourself/your skills; Knowledge of the application process; Exposure to/knowledge of life as a doctor; Mentorship/support; Clinical placements/shadowing; Other (please specify)
5	What did you find most useful in this programme? Why?	Free-text response
6	How could we improve the programme?	Free-text response
7	What further support would be most beneficial? (Check all that apply)	Checkbox options: Ongoing mentorship; Guidance of useful resources; Work experience opportunities; Other (please specify)
